# Development of Germ Cell Isolation and Optimal Cryopreservation Method for *Lissachatina fulica* (*L. fulica*)

**DOI:** 10.3390/ani14223229

**Published:** 2024-11-11

**Authors:** Jukyeong Jeong, Seungki Lee, Jung Kyu Choi

**Affiliations:** 1 Department of Biotechnology, College of Life and Applied Sciences, Yeungnam University, Gyeongsan 38541, Republic of Korea; jjk1016jjk@naver.com; 2 Biological and Genetic Resources Assessment Division, National Institute of Biological Resources, Incheon 22689, Republic of Korea

**Keywords:** ovotestis, vitrification, *Lissachatina fulica*, sperms

## Abstract

This study aims to develop an optimized method for cryopreserving the germ cells of *Lissachatina fulica* (*L. fulica*) using vitrification, as an alternative approach for conserving endangered snail species. First, we isolated several key reproductive organs, including the sperm oviduct, albumen gland, hermaphrodite gland (ovotestis), and hermaphrodite duct from *L. fulica*. When the ovotestis was finely chopped, numerous sperm with long tails and distinct heads were observed. The staining of sperm nuclei was confirmed using Hoechst 33342 dye. Since the hermaphrodite gland, referred to as the ovotestis, contains both male and female germ cells, we performed tissue staining on the ovotestis using hematoxylin and eosin (H&E) dye. H&E staining of the ovotestis revealed numerous oval-shaped acini containing sperm and early germ cells. Spermatocytes and spermatids were observed within distinct boundaries, with mature sperm appearing following spermatogenesis. To preserve the species of the *L. fulica*, we introduced vitrification technology to cryopreserve its reproductive organs. The non-vitrification group showed an average cell viability of 96.6%, while the vitrification group had 86.8% after thawing. This study presents a reliable cryopreservation protocol for *L. fulica*, with potential applications for other endangered snails, supporting conservation efforts to preserve genetic resources and biodiversity.

## Simple Summary

A cryopreservation method for *L. fulica* germ cells was developed using vitrification. Key reproductive organs, including the ovotestis containing both male and female germ cells, were isolated. Vitrification technology was applied, resulting in 86.8% cell viability post-thawing compared to 96.6% in the non-vitrified group. This study establishes a reliable protocol for preserving *L. fulica* germ cells, with potential applications for conserving other endangered snail species.

## 1. Introduction

*Lissachatina fulica* (*L. fulica*), known as the giant African snail, is widely recognized around the world as a significant snail pest, causing agricultural and ecological damage due to its extensive dietary habits and high reproductive rate [1,2]. However, *L. fulica* can positively contribute to biodiversity in ecosystems and scientific research, including neurobiology, physiology, ecology, and biomedical studies [3]. From a biodiversity perspective, *L. fulica* plays a role in ecosystem maintenance by decomposing organic matter, dispersing seeds, and providing a food source for various predators [4]. In terms of scientific research, *L. fulica* serves as a valuable model for investigating neural circuits, physiological responses to environmental stress, and ecological interaction [5,6]. In medical research, compounds derived from *L. fulica* exhibit antimicrobial, anti-inflammatory, and wound-healing properties, offering potential for new treatment developments [7,8].

Despite extensive studies on *L. fulica*, its reproductive research remains largely unexplored. The reproductive system of *L. fulica* functions as simultaneous hermaphrodites, featuring ovotestes that contain both male germ cells, like spermatogenic cells, and female germ cells, like ova, at the same time [9]. *L. fulica*, with its large size and high reproductive rate, serves as an ideal model for studying snail reproduction, providing an alternative to endangered snail species, which are challenging to study due to difficulties in obtaining sufficient specimens. Ecological destruction, soil pollution, and the introduction of invasive species have led to a decline in the original snail populations in their habitats, resulting in their addition to the list of endangered species of concern [10].

In this study, we isolated reproductive cells from the ovotestis of *L. fulica* as a snail model for conserving other endangered snail species and confirmed their presence in the ovotestis using H&E staining. Next, we developed an optimized vitrification cryopreservation system for the reproductive cells of *L. fulica*. In a previous study, the survival of germ cells in the ovotestis of Oriental snails after freeze-thawing was confirmed using live/dead staining [11]. However, in this study, we quantitatively analyzed the results of live/dead staining after subjecting the ovotestis of African giant snail to the same treatment. Therefore, the findings present a cryopreservation protocol for *L. fulica*, with the potential applications for other endangered snails, thereby supporting conservation efforts to protect vital genetic resources and biodiversity.

## 2. Materials and Methods

### 2.1. Habitat Conditions for Lissachatina fulica (L. fulica)

A total of 32 giant African snails were utilized in this study. *L. fulica* were kept in an environment with 14 h of light and 10 h of darkness. They were housed in a plastic breeding container measuring 34 cm in length, 20.5 cm in width, and 25 cm in height. The bottom of the container was lined with about 3 cm of cocopeat, which is a type of soil specifically for snails. To maintain a humid environment, moist moss was provided. The snails, which are omnivorous, were fed a mixed diet combined with a calcium supplement dissolved in water, along with vegetables such as carrots to supplement their nutrition. The food was replaced every 2–3 days, and a spray bottle was used to mist water twice a day to maintain humidity. The temperature was maintained between 24 and 28 °C. The snails used in the experiment had a shell length of approximately 8 cm and a body length of about 20 cm.

### 2.2. Isolation of Reproductive Organs from L. fulica

The protocols for euthanasia and dissection of *L. fulica* were adapted from the methods described in [12]. To minimize the pain experienced by the snails, anesthesia was administered before euthanasia. Initially, anesthesia was conducted using 5% ethanol, followed by euthanasia with 70% ethanol. The snails were placed in a 250 mL beaker containing 5% ethanol, which resulted in the release of feces, mucus, and air bubbles from the anus. After 30 min, the absence of a contraction response upon pinching the foot or skin with forceps confirmed the completion of anesthesia. Subsequently, euthanasia was carried out by treating the snails with 70% ethanol for 10 min. Using scissors, the shell of the snail was cut from the outside. After completely removing the shell, scissors and forceps were used to cut along the body line to apex and remove the outer membrane. The reproductive organs were then retrieved and transferred to a 35 mm petri dish containing 2 mL of DPBS. They were cut into 1 mm^2^ pieces using a 1 mL syringe for cryopreservation.

### 2.3. The Observation of Sperm from the Ovotestis and Identification of the Developmental Stages of Spermatozoa in the Ovotestis Using H&E Staining

To verify that the isolated reproductive organ was an ovotestis, we dissected the tissue and examined it under a microscope for the presence of sperm. The presence of a nucleus in the sperm head was confirmed by the strong fluorescent signal observed following Hoechst staining (1 μg/mL) for 5 min. The ovotestis were fixed overnight in 4% paraformaldehyde and subsequently embedded in paraffin blocks. Sections of 5 μm were cut using a microtome and treated with xylene and ethanol for dewaxing and hydration, followed by staining with hematoxylin and eosin. After staining, the sections were dehydrated, and a cover glass was applied for microscopic observation.

### 2.4. Cryopreservation and Cell Viability of Ovotestis from L. fulica

Ovotestis was placed on a copper grid using mouth pipettes and capillary tubes after being immersed for 10 min in L-15 based equilibrium medium containing 10% (*v*/*v*) dimethyl sulfoxide (DMSO), 10% (*v*/*v*) ethylene glycol (EG), and for 2 min in vitrification medium containing 15% (*v*/*v*) DMSO, 15% (*v*/*v*) EG, and 0.5 M sucrose. The tissue was then immersed in liquid nitrogen (LN_2_) for 1 min and sequentially thawed in L-15 based solutions containing 0.5 M, 0.25 M, 0.125 M, and 0 M sucrose for 3 min each.

The cells were isolated from minced tissues using a 1 mL syringe, and their viability was assessed using live/dead and nuclear staining techniques. Fluorescein diacetate (FDA) was used to stain live cells green, while propidium iodide (PI) (2 μg/mL) was used to stain dead cells red. The number of FDA-positive (green) and PI-positive (red) cells were quantified from the acquired images to determine cell viability.

### 2.5. Statistical Analysis

Statistical analysis was carried out with an analysis of variance (ANOVA) to determine the *p*-value across two groups (five times each). A *p*-value below 0.05 was regarded as indicative of a significant difference.

## 3. Results and Discussion

**Figure 1:**
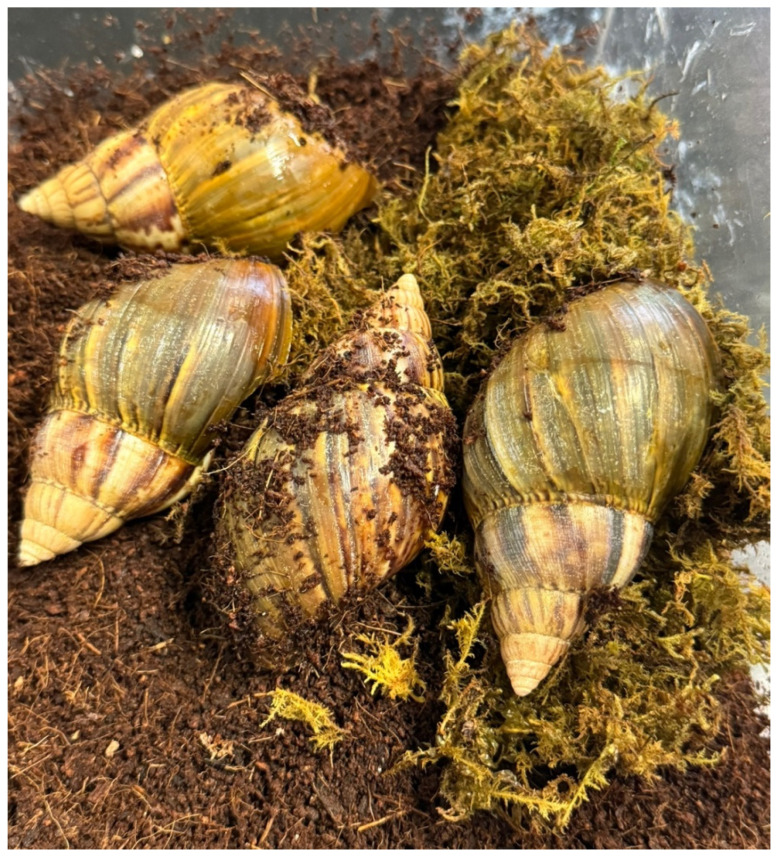
The environment of *Lissachatina fulica* (*L. fulica*). The habitant of *L. fulica* was carefully controlled to mimic its natural environment. The cage was maintained a constant humidity and a temperature range of 22–25 °C. The substrate of the cage consisted of a mixture of coconut coir and moss, which was kept moist to prevent dryness and to encourage burrowing behavior, but it was not waterlogged. The cone-shaped spire of *L. fulica* is mainly visible in hues of brown, gray, or a combination of the two.

As shown in Figure 1, due to the challenges in acquiring endangered snail species, we employed *L. fulica*, known for its high reproduction rate and significant size, as an alternative model for conservation efforts [1]. In studies focused on conserving endangered species, the technique of cryopreserving reproductive organs, including sperm and eggs, is widely recognized. This technology can increase the survival rate of germ cells and maintain the genetic diversity of endangered animals in the future, as well as increase populations through artificial breeding programs [11]. However, before freezing the reproductive organs of endangered snail species, we confirmed the location of the reproductive organs and the presence of reproductive cells in the ovotestis of the *L. fulica* through anatomical and histological analysis.

**Figure 2:**
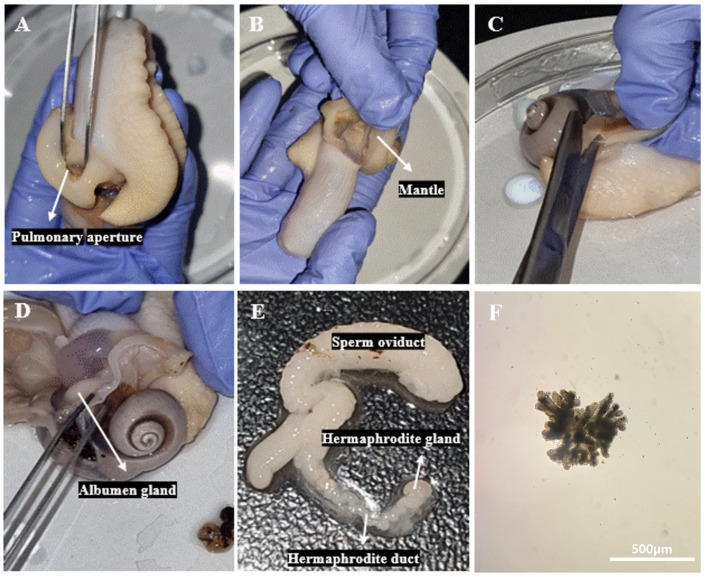
Isolation of reproductive organs from *L. fulica*. All dissection procedures were conducted following anesthesia with 5% ethanol and euthanasia with 70% ethanol. (**A**) Scissors were inserted through the pulmonary aperture. (**B**) The mantle was cut away from the body wall along the line of attachment, which was under the collar. (**C**) Another cut was made from the pulmonary aperture along the body towards the apex in the opposite direction. (**D**) When the mantle was lifted to the left, the albumen gland became visible. (**E**) The hermaphrodite duct and the hermaphrodite gland were discovered through the albumen gland. (**F**) The hermaphrodite gland was isolated from *L. fulica*. Scale bar = 500 µm.

The dissection of *L. fulica* uncovers several essential reproductive organs: the sperm oviduct, albumen gland, hermaphrodite gland, and hermaphrodite duct. The hermaphrodite gland, which generates both eggs and sperm, is noted to be connected to the hermaphrodite duct. The hermaphrodite duct carries the reproductive cells, while the albumen gland provides essential nutrients for egg development. Moreover, the sperm oviduct, which transports sperm, aids in fertilization by offering a route for sperm storage and movement towards the eggs (Figure 2A–E). The ovotestis, isolated for cryopreservation, was found to be composed of groups of oval-shaped acini, as indicated by the white arrow in Figure 2F.

**Figure 3:**
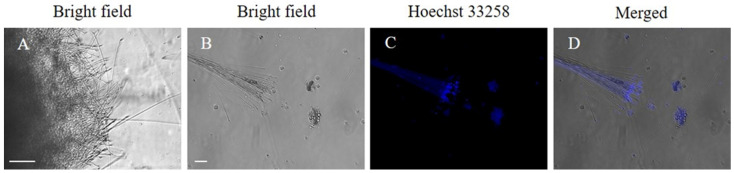
Isolating sperm from the ovotestis of *L. fulica.* (**A**) Sperm bundles were found in the ovotestis punched with a syringe. (**B**) The morphology of a bundle of sperm of *L. fulica* was examined under a microscope after staining with Hoechst 33258 (nuclear staining). Nuclei (blue) were distinctly observed within the heads of the sperm (**C**,**D**). Scale bar = 50 µm.

**Figure 4:**
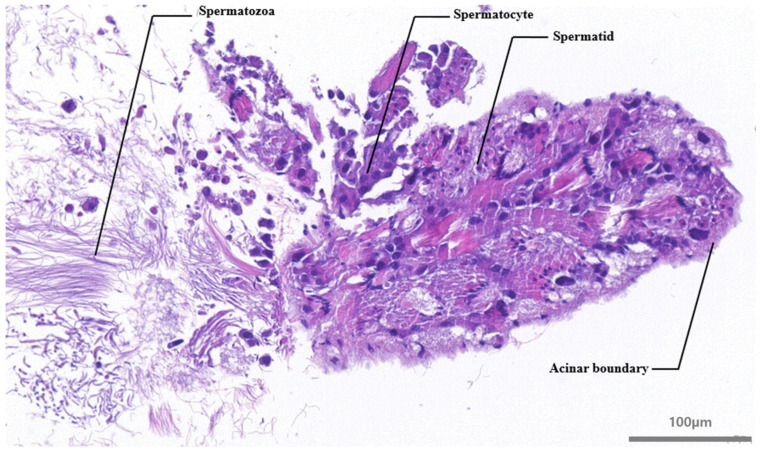
Histology of ovotestis of *L. fulica*. The ovotestis was observed to consist of numerous oval-shaped acini, within which spermatocytes, spermatids, and sperm were clearly identified. Spermatocytes displayed irregular shapes and were larger in size, characterized by prominent nuclei. Spermatids were positioned closer to the lumen compared to spermatocytes and exhibited denser nuclei. Following spermatogenesis, mature sperm with tails were observed within the acini. Scale bar = 100 µm.

Sperm were found in the ovotestis of *L. fulica*, which was finely minced to release the sperm cells, uncovering numerous sperm bundles. The morphology of the sperm, which exhibited a typical head and a long tail, was observed. Staining with Hoechst 33258 facilitated clear visualization of the nuclei within the heads of the sperm (Figure 3). H&E staining of the ovotestis revealed numerous oval-shaped acini containing sperm and early germ cells. Spermatocytes and spermatids were observed within distinct boundaries, with mature sperm appearing following spermatogenesis (Figure 4). We successfully established a method for isolating the ovotestis and sperm cells of *L. fulica*. The sperm morphology, characterized by a typical head and a long tail, was observed in vitro and the presence of a nucleus in the head of the snail sperm was confirmed using Hoechst 33258 staining. Moreover, we successfully identified the presence of sperm cells and their developmental stage within the tissue using H&E staining on the ovotestis. For snails, which are hermaphrodites, it is expected that both sperm and eggs will be found in the ovotestis [13]. The absence of oocytes in the African snail specimens examined in this study can be attributed to the immaturity of the individuals, indicating a predominance of spermatogenesis over oogenesis [9]. In sexually mature snail, however, oogenesis is more prevalent. Thus, future studies will focus on long-term observations of snails of varying ages, employing gene expression profiling to elucidate the molecular mechanisms underlying oogenesis.

**Figure 5:**
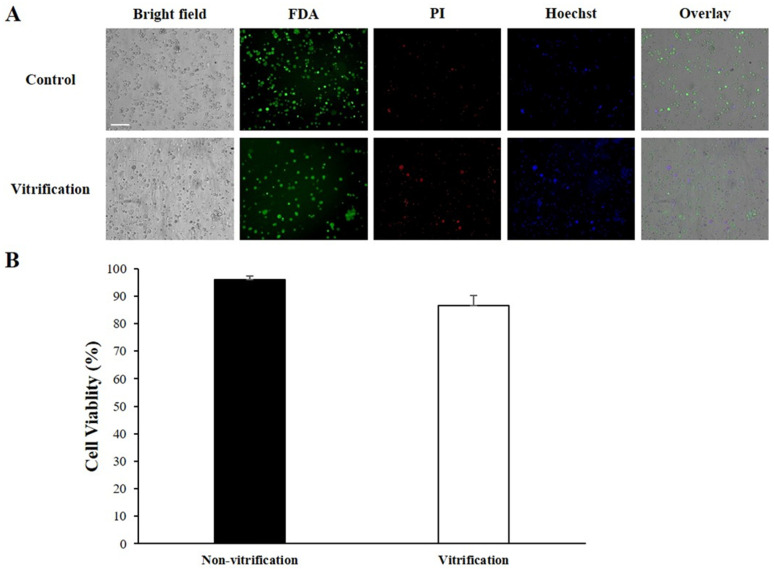
Cell viability and quantification of reproductive cells in the ovotestis of *L. fulica* after vitrification. (**A**) Cell viability of reproductive cells in the ovotestis was validated by conducting evaluations using FDA staining for live cells (green) and PI staining for dead cells (red). (**B**) The figure demonstrates a significant difference (*p* = 0.05) in the number of viable reproductive cells following vitrification compared to the control (non-vitrification). Scale bar = 50 µm (**A**).

The viability of reproductive cells was evaluated following the vitrification of ovotestis of *L. fulica* using FDA and PI staining. Viable cells were marked with green fluorescence by FDA, whereas non-viable cells were marked with red fluorescence by PI (Figure 5A). The findings revealed that a substantial number of cells remained viable after thawing, highlighting the success of the vitrification method in maintaining cell viability. The control group (no freezing) showed an average cell viability of 96.1%, while the vitrification group, after undergoing vitrification and thawing, displayed an average cell viability of 86.4% (Figure 5B). This indicates a high survival rate of reproductive cells even after undergoing the vitrification process. Cryopreservation of snail ovotestis is still in its early stages of research. To assess the viability of the ovotestis post-freezing, we initially immersed the ovotestis in liquid nitrogen for 1 min followed by immediate thawing. In future studies, we plan to gradually increase the freezing duration to observe changes in viability. Firstly, we successfully preserved the ovotestis of *L. fulica* using vitrification techniques. After thawing, the viability of reproductive cells isolated from the ovotestis was found to reach up to 86.4% compared to the control (96.1%). Although there was a significant difference compared to the control (no freezing), this is the first study to provide quantitative data on the cryopreservation of reproductive cells in snails. However, to enhance the survival rate of snail reproductive cells post-cryopreservation, we plan to conduct research using the optimal concentration combinations of cell-penetrating cryoprotectants (CPAs) such as dimethyl sulfoxide (DMSO), ethylene glycol (EG), and propane-1,2-diol (PROH), along with non-penetrating cryoprotectants like sucrose and trehalose, which are commonly used for the cryopreservation of reproductive cells in other species such as mollusks [14], crustaceans [15], and fish [16]. Moreover, we propose to perform functional and genetic analysis of snail reproductive cells to evaluate the toxicity of CPAs both before and after thawing.

## 4. Conclusions

We successfully introduced a method for isolating and cryopreserving the ovotestis and sperm cells of *L. fulica*. Our results demonstrated that the viability of reproductive cells after vitrification remains high and provide the first quantitative data on snail reproductive cell cryopreservation. Therefore, this study not only provides fundamental data for reproductive research and species conservation of snails but also contributes to maintaining the genetic resources and diversity of endangered species.

## Data Availability

The data presented in this study are available on request from the corresponding author.
